# Tinker or Prophet?

**Published:** 1887-08-27

**Authors:** 


### TINKER OR PROPHET ?

"On earth there is nothing great but man: in man there is nothing great but mind." Most true; but mind works by means of the physical brain, and is strong or weak, courageous or fearful, resolute or yielding, according to the condition of the brain. He who teaches man to know, he who teaches man to hope, he who teaches man to do, he who teaches man to endure, each is great and worthy of that kind of gratitude which the world gives to those whom it holds in immortal remembrance. It is an unpleasing reflection to a doctor who loves man, and loves his art too, that the great world holds few of the dead of his own calling in affectionate memory. The monuments of physicians are built by physicians, their stories are told by professional writers to professional readers; and although they are not unhonoured, they are for the most part "unwept and unsung." Where is the physician, among all the learned Egyptians whom the world knows as it knows the man Moses? And yet at the time of Moses there were physicians of great fame and repute. What man who can take a measure of the world's judgments would compare Hippocrates with Paul? But Hippocrates has always had a name among the learned, whilst Paul was but the untiring missionary of a new and simple faith.

Medicine has almost always been a narrow art, concerning itself only or mainly with the sick. Is it of the essence of medicine that it be narrow? Is the physician merely the tinkerer of cracked kettles, the patcher-up of worn and wearing humanity? Is his business only with the sick; his message only to the diseased? Has he nothing to say to man as man? If the essence of man be mind: if that be the jewel and the body be only the casket: is the doctor merely a mender of caskets—he cannot pretend to be a maker of them? If that be so: if the physician be only the repairer of that which is the temporary accident of man, and have nothing at all to do with that which constitutes his real self and essence, then we can understand why for six thousand years the world has passed him by in silence; why it has raised no monuments to his honour; why it has sung no songs in his praise.

But has medicine now in these latter days anything to say to man whole, as well as to man sick? Its votaries have long been searching the deeper recesses of nature's mysteries. No digger for gold or jewels ever sought more eagerly in the bowels of the earth for costly treasures than have the labourers in the fields of medical science for true and veritable knowledge; knowledge which shall endure and be permanent when theories and speculations shall have changed and vanished away. Has all this labour been in vain? Must doctors still remain outside the real man and busy themselves with mending and patching his perishing outer shell? Has medicine no philosophy for the service of the learned; no evangel for the noble and enthusiastic lovers of their kind; nothing which the generous youth may lay hold of as he lays hold of a political theory or a religious faith, and carrying it to his fellows may say, "Here is light, here is hope, here is a human gospel which if you will but believe you shall find a great salvation"?

It cannot be denied that medicine has laboured long and with stern resolve to understand man. She has not laboured in vain. She has added something to the knowledge of the philosopher, something to the visions of the religious teacher. She has supplemented and enlarged; she has enlightened and softened. She has done and is doing a worthy part towards the victory of man over the material world, and the still greater victory of man over himself.

For strange and unlooked for as the result may be, the new gospel of medicine, like the noblest evangel of religion, is a gospel of self-denial; of stooping to conquer; of repressing the lower and the meaner self in order that the higher and nobler self may constantly enlarge and expand, until the perfect and typical physical and moral man is reached. Only with the perfection of physical health can the highest perfection of mental and moral health be attained. The man of noble mind may be poor in achievement because his mind is associated with a feeble and failing body. Medicine teaches him to use his powers with discrimination; to labour in such measure as strength will allow, and by the maintenance of his bodily health to secure for himself a lengthened period of life and labour, in order that the added time may compensate for the feeble efficiency.

But the great evangel of medicine is this, that man is one—bodily and mentally one; that there is no divorce between body and mind and soul; that the complete and typical man is he whose powers are all fully developed, and all as perfect as it is their nature to be. This is the kind of man who will be victorious in the conflict of life, who will do most in the service of his fellows, who will most perfectly accomplish the will of God. The uninstructed theologian has despised the body: the self-indulgent epicure has destroyed it: the wise teacher of a noble religion has proclaimed it "the temple of God": and the science of medicine of these later times has rediscovered the scientific verities of Paul, and proclaims that he who will be perfect must by understanding and by discipline, and according to the fullest measure which is possible to him, be perfect in body as well as in mind and soul.

Is then the modern doctor a mere mender of bones and muscles, of nerves and blood-vessels, of livers and lungs; or is he a prophet and daily teacher of those principles of sense and health which in the present complex civilisation are not of secondary, but of primary importance to man? If he understands at all his own work in life, he is emphatically a teacher and a prophet. The grammarian teaches the art of speech; but without the tongue there is no instrument which can use it; the logician teaches the management of the reasoning powers; but apart from the brain where is the reasoner to be found? And so the doctor is a teacher, not of the use of one organ or one faculty merely, but of the perfect use of every organ and every faculty of the whole man, body, soul, and spirit.

If it be said, "There are few physicians who take this view of their functions; and fewer still who carry out their theories in their daily practice," the answer is twofold. In the first place the world has not yet, to any great extent, accepted the medical man as a teacher; and secondly, there is now arising a new school of physicians, who see the greatness of their own calling as teachers: men who are not content to be kettle-menders; who have vision and insight; who see what it is possible for man to become in the course of ages; men who are not to be silenced and quenched by the blind human moles who perceive nothing beyond their own little life and that of their own generation. The new teachers may be a few years, or a few scores of years, before their time. What prophet has not known the experience of proclaiming his message to a stiff-necked and unbelieving generation? But that the scientific medicine which is already here, and which in the future will be present with us more and more, has a teacher's and a prophet's, as well as a healer's function, no one can doubt whose eyes are opened to the movements of the times. Whosoever is wise will do his utmost to profit by the teaching.

